# Highly Sensitive Aluminum-Based Biosensors using Tailorable Fano Resonances in Capped Nanostructures

**DOI:** 10.1038/srep44104

**Published:** 2017-03-08

**Authors:** Kuang-Li Lee, Hsuan-Yeh Hsu, Meng-Lin You, Chia-Chun Chang, Ming-Yang Pan, Xu Shi, Kosei Ueno, Hiroaki Misawa, Pei-Kuen Wei

**Affiliations:** 1 Research Center for Applied Sciences, Academia Sinica, 128, section 2, Academia Road, Nangkang, Taipei 11529, Taiwan; 2 Institute of Optoelectronic Sciences, National Taiwan Ocean University, Keelung 20224, Taiwan; 3 Institute of Photonics Technologies, National Tsing Hua University, Hsinchu 30013, Taiwan; 4 Research Institute for Electronic Science, Hokkaido University, Hokkaido 060-0808, Japan; 5 Department of Applied Chemistry & Institute of Molecular Science, National Chiao Tung University, Hsinechu 20010, Taiwan; 6 Institute of Biophotonics, National Yang-Ming University, Taipei 11221, Taiwan

## Abstract

Metallic nanostructure-based surface plasmon sensors are capable of real-time, label-free, and multiplexed detections for chemical and biomedical applications. Recently, the studies of aluminum-based biosensors have attracted a large attention because aluminum is a more cost-effective metal and relatively stable. However, the intrinsic properties of aluminum, having a large imaginary part of the dielectric function and a longer evanescent length, limit its sensing capability. Here we show that capped aluminum nanoslits fabricated on plastic films using hot embossing lithography can provide tailorable Fano resonances. Changing height of nanostructures and deposited metal film thickness modulated the transmission spectrum, which varied from Wood’s anomaly-dominant resonance, asymmetric Fano profile to surface plasmon-dominant resonance. For biolayer detections, the maximum surface sensitivity occurred at the dip of asymmetric Fano profile. The optimal Fano factor was close to −1.3. The wavelength and intensity sensitivities for surface thickness were up to 2.58 nm/nm and 90%/nm, respectively. The limit of detection (LOD) of thickness reached 0.018 nm. We attributed the enhanced surface sensitivity for capped aluminum nanoslits to a reduced evanescent length and sharp slope of the asymmetric Fano profile. The protein-protein interaction experiments verified the high sensitivity of capped nanostructures. The LOD was down to 236 fg/mL.

Surface plasmon resonance (SPR) sensing is capable of sensitive, real-time and label-free detection for many applications, such as medical diagnostics, environmental monitoring and food safety^1^,^2^,^3^,^4^. Commercial sensing platforms utilize attenuated total reflection (ATR) method to induce the propagation of surface plasmon polaritons on thin noble metals and to enable real-time and label-free measurements of biomolecular binding affinity. Metallic nanostructure-based SPR sensors offer an alternative method for SPR excitation and have been used for biosensing applications^5^,^6^,^7^,^8^,^9^,^10^,^11^,^12^. Compared to prism-based SPR sensors, metallic nanostructures have a number of benefits, including their small detection volume, simple measurement and ease of multiple detections. However, the majority of SPR sensors utilize noble metals such as Au and Ag because these materials have low optical losses in the visible and near-infrared ranges. The Au material is commonly used for the sensors because of its high chemical stability. On the other hand, the Ag is easy to oxidize and unstable but exhibits more narrow and intense plasmon resonances. To prevent the Ag film from oxidation, a passivation dielectric film, such as silica^13^ or alumina^14^, is utilized. However, the high cost of these metals limits large-scale commercialization of plasmonics sensors. Recently, the studies of nanostructure-based aluminum sensors have attracted a large attention because aluminum is a more cost-effective material and relatively stable. Various nanostructure-based aluminum biosensors such as nanoconcave arrays^15^, nanohole^16^,^17^,^18^ and triangular nanoparticles^19^ have been proposed. To widely used aluminum material for sensing, the challenges from oxidation and material degradation (corrosion and pitting) have to be faced. These issues can be addressed by depositing a passivation dielectric film or using a passivation treatment based on oxygen plasma to produce an oxide protecting layer^17^. However, the large imaginary part of the dielectric function for aluminum results in a broad resonance response and a longer surface evanescent field, which limit its surface sensing capability. Therefore, improving surface sensitivities of nanostructure-based aluminum sensors is an important issue.

To evaluate the quality of a sensor, figure of merit (FOM) values are utilized. One of the FOM values, in wavelength units, is defined as *S*_*λ*_ (nm/RIU)/*fwhm* (nm), where *S*_*λ*_ is the linear regression slope for the refractive index dependence (bulk sensitivity), and *fwhm* is the resonance width of the plasmon resonance^20^. The redshift of the resonance wavelength, caused by a small index change, *Δn*, also induces a relative intensity change, *ΔI*/*I*, at a fixed wavelength near the resonance condition. An alternative FOM* value is proposed and defined as^21^,^22^


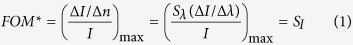


The FOM* value or intensity sensitivity (*S*_*I*_) is proportional to the bulk sensitivity and slope of the spectrum. On the other hand, to evaluate the surface sensing capability of a biosensor, the quantity FoM_t_ is used instead of FoM* for the thickness measurement^23^. FOM_t_ is defined as


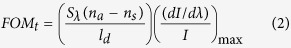


where *n*_*s*_ is the bulk solution refractive index, *n*_*a*_ is the adsorbate monolayer refractive index and *l*_*d*_ is the length of surface evanescent field. The surface sensitivity is determined by bulk sensitivity, evanescent decay length, and refractive index difference between the adsorbate monolayer and surrounding environment, and the resonant slope. For the surface plasmon wave propagating on a flat metal surface, the *l*_*d*_ (where the amplitude drops to 1/e) is determined primarily by the resonance wavelength λ and can be express as follows^1^:


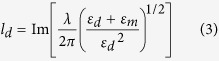


where *ε*_*m*_ and *ε*_*d*_ are the relative permittivities of the metal and the adjacent dielectric material. For periodic metallic nanostructures, the bulk sensitivity is increased with a longer period^24^,^25^. However, the decay length is also increased. The most efficient way to enhance the surface sensitivity is to reduce the resonant linewidth, a thermal-annealing method is used to produce high-quality metallic nanostructures. The annealed nanostructures have smoother metal surfaces and larger gold grains^13^,^25^,^26^,^27^,^28^, which reduce surface plasmon propagation loss and result in a sharp linewidth. Another approach to achieve a sharp spectral response is based on Fano resonances^29^,^30^,^31^. The Fano resonance exhibits a distinctly asymmetric shape, which arises from the spectral overlap between a broad resonance and a narrow discrete resonance^30^. The Fano resonances have been extensively studied in nanoparticles^5^, plasmonic nanostructures^9^,^31^ and metamaterials^32^.

In this paper, we utilized hot embossing nanoimprint lithography and thermal evaporation to fabricate low-cost, large-area, and highly sensitive aluminum nanostructures on A4 size plastic films. The optical properties, bulk and surface sensitivities of these aluminum nanostructures with different Fano resonance factors were studied and compared using wavelength and intensity interrogation. In addition, the sensing capabilities of the proposed nanostructures were verified by measuring interactions between bovine serum albumin (BSA) and anti-BSA. We found capped aluminum nanoslits exhibited obvious modulation of Fano resonances by changing the ridge height of nanostructures and the deposited metal film thickness. The transmission spectrum changed from Wood’s anomaly-dominant (peak) resonance, asymmetric Fano profile (peak and dip) to SPR-dominant (dip) resonance. The corresponding Fano factors extracted from the resonant profiles ranged from ~∞ to −0.12. The Fano resonance degenerates to a pure SPR (Lorentzian resonance) can be observed. For a transverse-magnetic (TM) polarized wave in capped aluminum nanoslits with a period of 470 nm, the narrowest bandwidth of the Fano resonance was only 2.7 nm. As the proposed nanostructure has an extremely sharp resonance in the visible light region, it reaches a figure of merit of 150 and an intensity sensitivity up to 29,345%/RIU (refractive index unit). The Fano resonance also plays an important role on the surface sensitivity. Our results show that the sensitivity at dip wavelength was much higher than sensitivity at peak wavelength. When the Fano factor was close to −1.3, the dip wavelength sensitivity was 2.58 nm/nm, which was about 2.6 times higher than that of Lorentzian resonance. In addition, the maximum intensity sensitivity for thickness was 90%/nm and the limit of thickness detection was 0.018 nm. We attributed the improved surface sensitivity for capped aluminum nanoslits with typical Fano resonances (q = −1.3) to a reduced decay length and sharp resonance slope. The protein-protein interaction experiments verified the high thickness sensitivity of the structures and a limit of detection (LOD) of 236 fg/mL anti-BSA was achieved.

## Results

### Optical properties of the capped aluminum nanoslits

**Figure 1:**
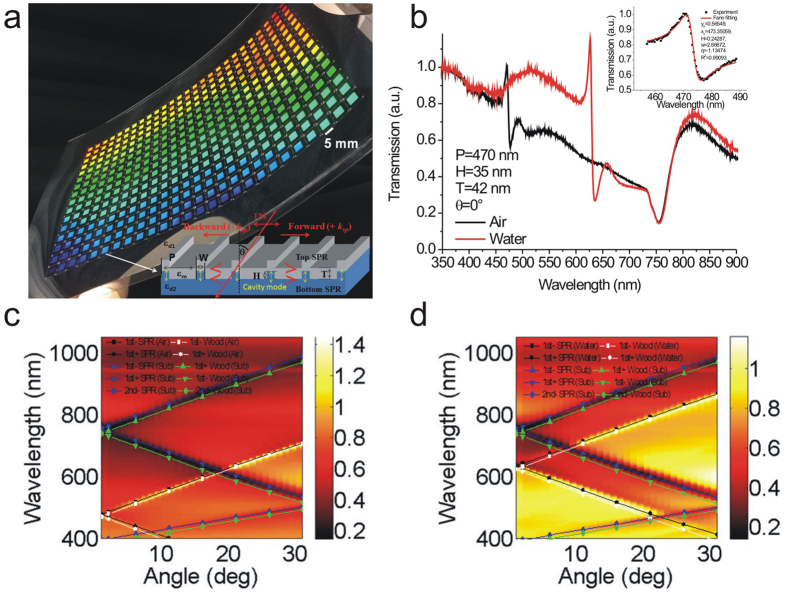
Optical properties of the capped aluminum nanoslits. (**a**) The optical image of the capped aluminum nanoslit arrays on an A4 size polycarbonate film. There are 416 arrays and the area of each periodic nanostructure is 5 × 5 mm^2^. The inset shows a schematic configuration depicting the geometrical parameters of the capped aluminum nanoslits and the direction of the transverse magnetic (TM)-polarized incident light. (**b**) Measured transmission spectra of the 470-nm-period capped aluminum nanoslits in air and water for normally incident TM-polarized light. We chose H = 35 nm, T = 42 nm, W = 60 nm, and P = 470 nm for the structure. The inset shows the Breit-Wigner-Fano fitting curve of the Fano resonance in air environment. According to the fitting equation of the Fano resonance, the linewidth (*w*) and Fano factor (*q*) was 2.7 nm and −1.13, respectively. (**c**,**d**) The measured angular transmission diagram of 470-nm-period capped nanoslit arrays with a 42-nm-thick aluminum film in air (**c**) and water (**d**) for TM-polarized incident light, respectively. The solid lines show the theoretical resonance wavelengths (calculated using eqs (4) and (5)) for the SPR modes and Wood’s anomaly, respectively.

Figure 1a shows the optical image of the capped aluminum nanoslit arrays on an A4 size polycarbonate film. There are 416 arrays and the area of each periodic nanostructure is 5 × 5 mm^2^. The inset shows a schematic configuration depicting the geometrical parameters of the capped aluminum nanoslits and the polarization direction of incident light. Figure 1b shows the measured transmission spectra of 470-nm-period capped aluminum nanoslits in air and water for normally incident TM-polarized light. We chose H = 35 nm, T = 42 nm, W = 60 nm, and P = 470 nm for the nanostructure. There are transmission peaks and dips in the spectrum due to the couplings of cavity resonances in nanoslits and surface waves on both sides of the periodic aluminum surface (the aluminum/medium and aluminum/substrate interfaces). The gap plasmons transmit through the nanoslits and capping layer, leading to a broadband transmission within a cavity spectrum. The resonance condition can be estimated using a Fabry-Perot cavity^33^. The resonance wavelength is determined by the gap width and cavity length. On the other hand, the cavity mode is coupled to the surface waves from the edges of the top and bottom interfaces. There are two kinds of surface waves for periodic metallic structures. For continuous metallic film, Bloch wave surface plasmon polariton (BW-SPPs) occurs when the Bragg condition is satisfied. The Bragg condition for one-dimensional arrays can be described by^1^


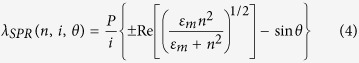


where *i* is the resonance order, *P* is the period of the nanostructure, *θ* is the incident angle, and *n* is environmental refractive index. The cavity mode is coupled to the BW-SPP mode and results in a transmission dip. On the other hand, the long-range SPP cannot exist for non-continuous film. The re-distribution of diffracted photons results in the Wood’s anomaly and a transmission peak happens under the condition,


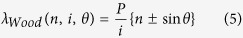


When the periodic metallic structures has a surface close to critical condition of continuous/isolated metallic film, the Wood’s anomaly and BW-SPPs play together with cavity mode. The interaction between cavity resonances in nanoslits (a continuum state) and Wood’s anomaly/BW-SPPs (a discrete resonance state) creates a Fano resonance profile consisting of a minimum (dip) and an adjacent maximum (peak)^30^,^34^,^35^. It generates a sharp resonance profile. In the case of capped nanoslit arrays with a 470-nm-period, extremely sharp Fano resonances were observed. The resonance dip wavelengths of Fano resonances at the air/aluminum and substrate/aluminum interfaces were 477 and 755 nm, respectively. Such resonances can be fitted by the Fano resonance equation (Breit-Wigner-Fano line shape),


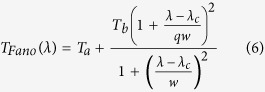


where *T*_*a*_, *λ*_*c*_, *T*_*b*_, and *w* are standard parameters that denote a slowly varying transmittance, the resonance wavelength, the contribution of a zero-order continuum state that couples with the discrete resonance state and the linewidth of the resonance, respectively. *q* is the Breit-Wigner-Fano parameter which describes the coupling strength. According to the fitting equation of the Fano resonance at the air/aluminum interface, the linewidth (*w*) and Fano factor (*q*) was 2.7 nm and −1.13, respectively (see Fig. 1b inset). To the best of our knowledge, it is the narrowest bandwidth observed in aluminum nanostructures. When the array was covered with water, the Fano resonance dip at the air/aluminum interface was redshifted to a wavelength of 635 nm and the resonance dip at the substrate/aluminum interface remained unchanged. Figure 1c and d show the measured angular transmission diagram of 470-nm-period capped nanoslit arrays with a 42-nm-thick aluminum film in air and water for TM-polarized incident light, respectively. The solid lines show the theoretical resonance wavelengths (calculated using eqs (4) and (5)) for the SPR modes and Wood’s anomaly, respectively. The wavelength dependence permittivity of aluminum is obtained from Rakić^36^. The experimental results were close to the calculated values.

**Figure 2:**
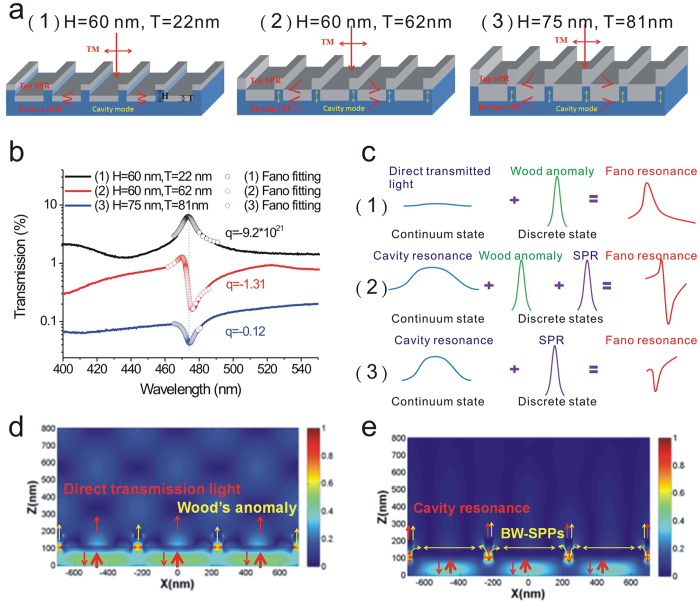
The mechanism and measurement of Fano resonances in different capped aluminum nanoslit arrays. (**a**) A schematic configuration, depicting the geometrical parameters of the capped nanoslits with various ridge heights (H = 60–75 nm) and aluminum film thicknesses (T = 22–81 nm). (**b**) The measured transmission spectra of these capped aluminum nanoslits for TM-polarized incident light. The open circles show the Fano fittings for different nanostructures. The Fano factors are −9.2 × 10^21^, −1.31 and −0.12, respectively. (**c**) A schematic illustration demonstrates the Fano resonances with different Fano factors in these nanostructures. (**d**) The resonance field (Ex) distribution at the resonance peak for 20-nm-thick capped aluminum nanoslits. (**e**) The Ex field distribution at Fano resonance dip for 60-nm-thick capped aluminum nanoslits.

It was noted that the Fano profile is modulated by the linewidth and the ratio between the resonance transition amplitude and the direct transition amplitude^37^. Figure 2a shows a schematic configuration, depicting the geometrical parameters of the capped nanoslits with various ridge heights (H = 60, 60, and 75 nm) and aluminum thicknesses (T = 22, 62, and 81 nm). The measured transmission spectra of these capped aluminum nanoslits were shown in Fig. 2b. Figure 2c shows a schematic illustration demonstrates the Fano resonances with different Fano factors in these nanostructures. For the 22-nm-thick nanostructure, it was too thin to form continuous film on 60 nm-height grooves. The direct transmission through metal film and Wood’s anomaly contribute to the transmission spectrum. A resonance peak was observed in the transmission spectrum. The Fano fitting indicates that the Fano factor (q_1_) is −9.2 × 10^21^, i.e. ~∞. When the thickness increases to 62 nm, the film thickness is close to 60 nm-height grooves. It excited cavity resonance, BW-SPP resonance and Wood’s anomaly. The coupling of these three modes lead to a sharp asymmetric resonance profile (peak and dip) with a Fano factor, q_2_ = −1.31. When the thickness T increased to 81 nm, the metallic surface became continuous. As a consequence, a SPR dip was observed in the transmission spectrum and the Fano factor increased to −0.12 (q_3_). The Fano resonance gradually degenerates to a Lorentzian resonance, i.e. q = 0. To verify the effect of the metal thickness on the continuum states, we utilized finite-difference time-domain (FDTD, FullWAVE 4.0, RSoft) simulations to study the differences of the forming continuum states in 20 and 60-nm-thick capped aluminumnanoslits. As the cavity resonance can only be excited with a TM polarized wave^38^, we studied the optical field (Ex) distributions of the resonances. Figure 2d and e show the resonance field (Ex) distributions for the resonance peak and Fano resonance dip for 20 and 60-nm-thick capped aluminum nanoslits, respectively. For 20-nm-thick capped aluminum nanoslits, the far field distribution was caused by the interference between the direct transmission light through metal film (a continuum state) and Wood’s anomaly. For 60-nm-thick nanostructures, the direct transmission light was reduced. The light propagates through the metallic nanoslit via gap plasmon, which forms the cavity resonance due to the reflectance at upper and lower interfaces. The cavity mode is a broadband resonance. It radiates from the edge and couples to the BW-SPP resonance. With the capped metal above the nanoslits, the coupling between the cavity and BW-SPP resonances was enhanced. Therefore, the cavity and BW-SPP resonances contributed to the far field distribution.

### Refractive index sensing capabilities of the capped aluminum nanoslits

**Figure 3:**
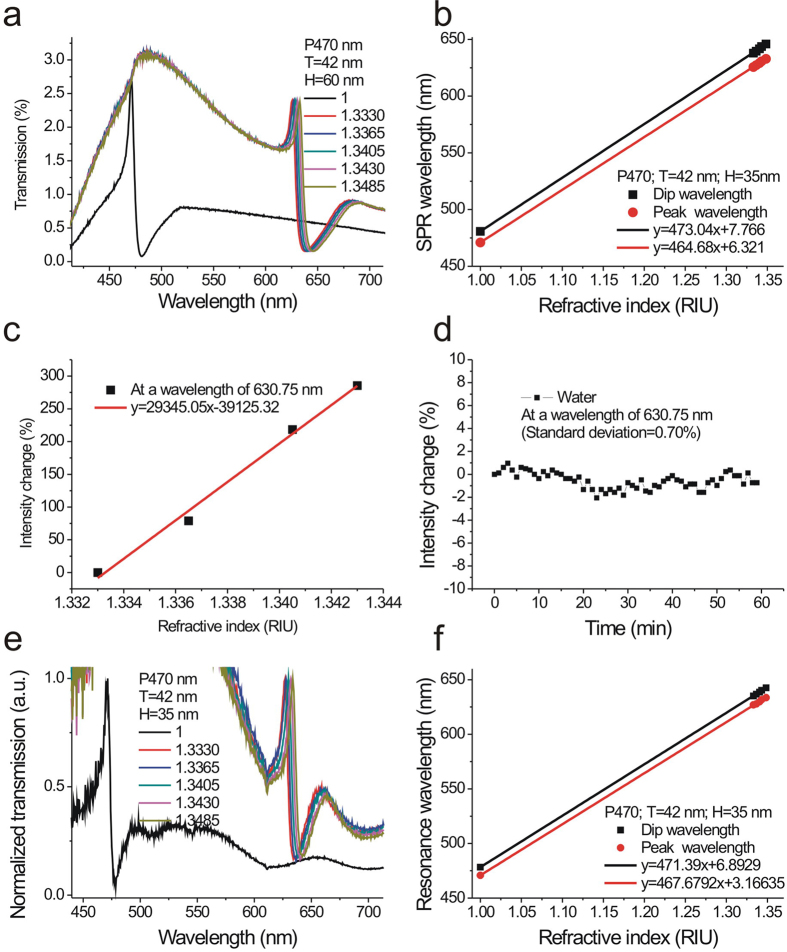
Refractive index sensing capabilities of the capped aluminum nanoslits. (**a**) The transmission spectra of the capped aluminum nanoslits with various water/glycerin mixtures for a normally-incident TM-polarized wave. The structure parameters are H = 60 nm, T = 42 nm, W = 60 nm, and P = 470 nm. (**b**) The peak wavelength and dip wavelength against the refractive index of outside medium. The slopes of the fitting curves show that the bulk sensitivities were 464 and 473 nm/RIU for the resonance peak and dip, respectively. (**c**) The normalized intensity change against the refractive index of the outside medium. The slope of the fitting curve shows that the intensity sensitivity was 29,345%/RIU. (**d**) The intensity change as a function of time at a wavelength of 630.75 nm. (**e**) The transmission spectra of the capped aluminum nanoslits with various water/glycerin mixtures for a normally-incident TM-polarized wave. The structure parameters are H = 35 nm, T = 42 nm, W = 60 nm, and P = 470 nm. (**f**) The peak wavelength and dip wavelength against the refractive index of outside medium for the nanostructure with a ridge height of 35 nm. The peak and dip wavelength sensitivities were 467 and 471 nm/RIU, respectively.

To verify the sensitivity of the capped aluminum nanoslits, we first tested the refractive index sensitivities of the nanostructures with a period of 470 nm. The bulk sensitivity of the sensor was measured by injecting purified water mixed with various ratios of glycerin into the microfluidic devices. Figure 3a shows the transmission spectra of the capped aluminum nanoslits with various water/glycerin mixtures for a normally-incident TM-polarized wave. The structure parameters are H = 60 nm, T = 42 nm, W = 60 nm, and P = 470 nm. There were sharp Fano resonances in the spectra. The peak and dip wavelengths of the Fano resonance at the air/metal interface were 471 and 480 nm, respectively. When the concentrations of glycerin/water mixtures increased, the wavelengths of Fano resonances were red-shifted and the intensities were changed. Figure 3b shows the peak wavelength and dip wavelength against the refractive index of outside medium. The slopes of the fitting curves show that the RIU (refractive index unit) sensitivities were 464 and 473 nm/RIU for the resonance peak and dip, respectively. These sensitivities are close to the theoretical sensitivity which indicates that the wavelength sensitivity (*S*_*λ*_) is close to the period of the nanostructures, i.e. *S*_*λ*_ ~ *P* nm/*RIU*. Figure 3c shows the normalized intensity change against the refractive index of the outside medium. The slope of the fitting curve shows that the intensity sensitivity was 29,345%/RIU. The measured intensity sensitivity is much better than the reported intensity sensitivities of gold nanoslit, nanohole or nanogrid arrays ~1,010–12,963%/RIU^27^,^39^,^40^ and ATR-based SPR sensors ~ 15,000%/RIU^2^. For the current system, the integration time for acquiring one spectrum was 25 milliseconds and the intensity noise was 0.7%. Thus, the detectable refractive index resolution was 2.38 × 10^−5^ RIU. If the intensity stability is further reduced to 0.2%, the structure can achieve a bulk refractive index resolution of 6.8 × 10^−6^ RIU. Such a sensitivity is comparable with commercial SPR machines using complicated high-resolution angular detection method. To compare the refractive index sensing capability of the fabricated nanostructures with previous works, we also calculated the figure of merit (FOM) values in wavelength units. The measured bandwidth of the Fano resonant peak in air (n = 1) was 3.1 nm and the wavelength sensitivity was 464 nm/RIU. Thus, the FOM value was 150 in the visible light region. The quality factor (resonant wavelength/line width) of the system was 152 (473 nm/3.1 nm). The obtained FOM is higher than that of the previously reported FOM in nanostructure-based aluminum sensors^15^,^16^,^17^,^41^,^42^, the FOM value (108) of the gold mushroom arrays^11^ and the theoretically estimated upper limits (FOM = 108) of the ATR-coupling SPR sensors^43^. We also tested the bulk sensitivity of capped nanoslits with different ridge height (H = 35) as shown in Fig. 3e and e. The results show that the peak and dip wavelength sensitivities were 467 and 471 nm/RIU, also close to the period of the nanostructures.

### Surface sensitivity tests for capped aluminum nanoslits with different Fano factors

**Figure 4:**
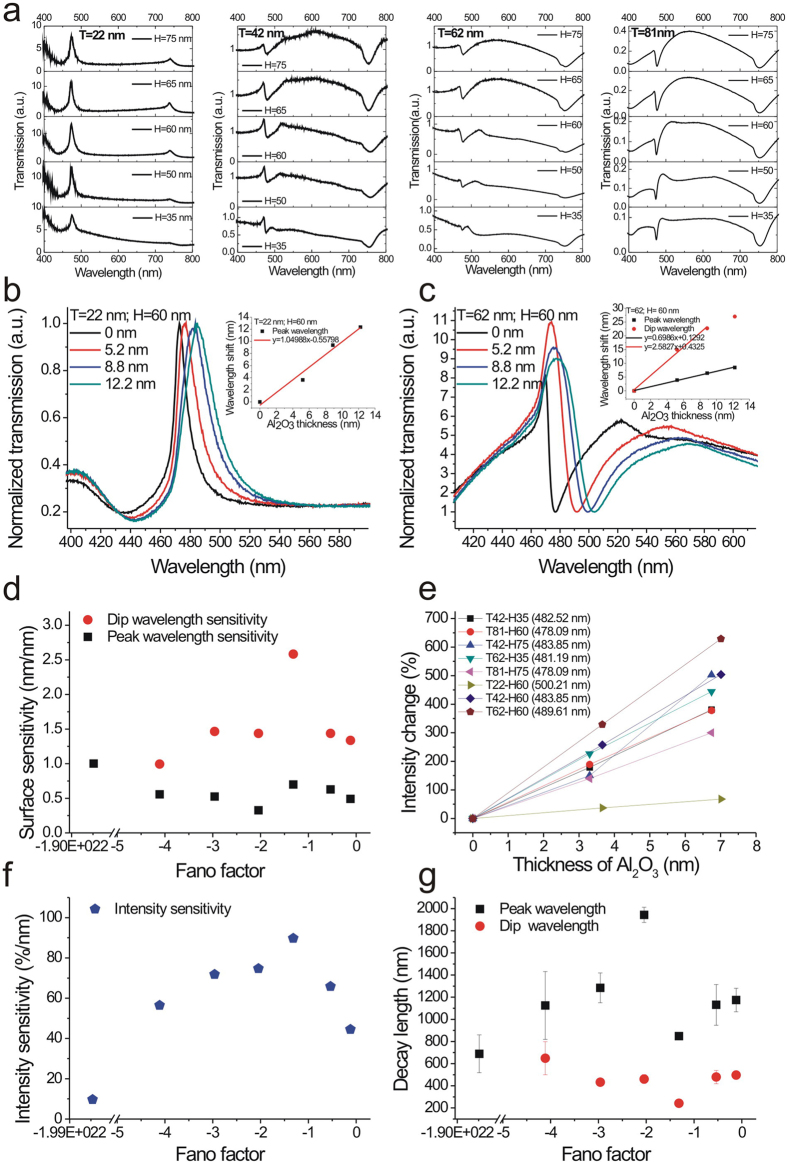
Surface sensitivity tests for capped aluminum nanoslits with different Fano factors. (**a**) The transmission spectra of capped aluminum nanoslits with different structure parameters in air for normally incident TM-polarized light. The structure parameters were P = 470 nm, H = 35–75 nm, T = 22–81 nm and W = 60 nm. (**b**,**c**) The spectra of nanostructures with ridge height of 60 nm and metal thicknesses of 22 (**b**) and 62 nm (**c**) for different Al_2_O_3_ film thicknesses. (**d**) The measured surface sensitivities for different Fano factors with wavelength interrogation. (**e**) The intensity change against the film thickness for different aluminum nanostructures. There were linear correlations between the intensity change and the film thickness. (**f**) The intensity sensitivity as a function of the Fano factor. The maximum intensity sensitivity (90%/nm) was obtained when the Fano factor was close to −1.3. (**g**) The calculated decay lengths at peak or dip wavelength for the capped nanoslits with different Fano factors.

For molecular binding events, the minimum detectable surface mass density is a key value to evaluate the quality of a sensor. We further compared the surface sensitivities of the nanostructures with different Fano factors by depositing different thicknesses of transparent thin films. Figure 4a shows the transmission spectra of capped aluminum nanoslits with different structure parameters. The structure parameters were P = 470 nm, H = 35–75 nm, T = 22–81 nm and W = 60 nm. The corresponding Fano factors can be obtained by Fano fitting curves. Figure 4b and c show the spectra of nanostructures with a ridge height of 60 nm and metal thicknesses (22 nm and 62 nm) for different thickness of Al_2_O_3_ film. The resonant peak or dip was redshifted with the increase of the film thickness. There were linear correlations between the peak or dip shift and the film thickness. The measured surface sensitivities for different Fano factors with wavelength interrogation were shown in Fig. 4d. The dip wavelength sensitivity was 1.7~5 times higher than peak wavelength sensitivity for different structures and dramatically increased when the Fano factor was close to −1.3. The dip wavelength sensitivities were 1.42, 2.58 and 0.99 nm/nm (wavelength shift/film thickness) for Fano factors of −0.53, −1.3 and −4.1, respectively. Compared to the sensitivities of Fano factors of −0.53 and −4.1, it was improved by a factor of 1.8 and 2.6, respectively. The enhanced sensitivity for capped aluminum nanoslits (2.58 nm/nm) is higher than that of Au nanohole arrays (2.08 nm/nm) and close to that of Ag nanohole arrays (2.60 nm/nm)^14^. With this sensitivity and the resolution of the spectrometer (0.1 nm), the capped aluminum nanoslits can detect a 0.04-nm-thick Al_2_O_3_ film deposited on the surface. In addition, we also compared the surface sensitivities of the nanostructures with intensity interrogation and analyzed the absolute spectral intensity changes (∆I/I_0_%) caused by the increased film thickness. Figure 4e shows the intensity change against the film thickness for different aluminum nanostructures. There were linear correlations between the intensity change and the film thickness. The intensity sensitivities were 56, 90, 65 and 9.6%/nm (intensity change/film thickness) for Fano factors of −4.1, −1.27, −0.53, and −9.2 × 10^21^, respectively. The maximum intensity sensitivity (90%/nm) was obtained when the Fano factor was close to −1.3 (See Fig. 4f). With 1.71% intensity change (3 times of noise level), the minimum detectable thickness was 0.018 nm.

It is noted that when an analyte is attached to the surface of plasmonic sensors, the effective refractive index (*n*_*eff*_) observed on the surface plasmon can be expressed as follows:


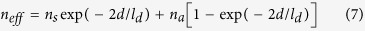


where *d* is the thickness of the adsorbate monolayer. The wavelength shift (Δλ) caused by the monolayer can be expressed as


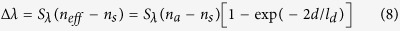


According to eq. (8), when the thickness of the analyte (d), the bulk solution refractive index (n_s_), and the adsorbate monolayer refractive index (n_a_) are chosen, the spectral shift is determined by the bulk sensitivity (*S*_*λ*_) and decay length (*l*_*d*_). In Fig. 3b and f, we have measured the bulk sensitivities of capped nanoslits with different structure parameters. The results show that bulk sensitivity is independent of parameters such as H and T and mainly determined by the period. The decay lengths for different nanostructures can be estimated from the measured bulk sensitivities (see Fig. 3b and f), wavelength shifts, thicknesses of the Al_2_O_3_ film, n_a_ = 1.67 and n_s_ = 1. Figure 4g shows the decay lengths for the capped nanoslits with different Fano factors. The results show that peak resonance has a longer decay length than the dip resonance. The mean values of the decay lengths are 1230 nm and 442 nm, respectively. The decay length of the dip resonance is close to the theoretical decay length (*l*_*d*_ = 421 nm, λ_SPR_ = 476 nm) for SPR on a flat aluminum surface. However, the dip resonance had a much shorter decay length when Fano factor was close to −1.3 (see Fig. 4g). The decay length was 242 nm for Fano factors of −1.31. Compared to the theoretical decay length, it reduced by a factor of 1.73.

### Simulations of transmission spectra and resonance field distributions of the capped nanoslits

**Figure 5:**
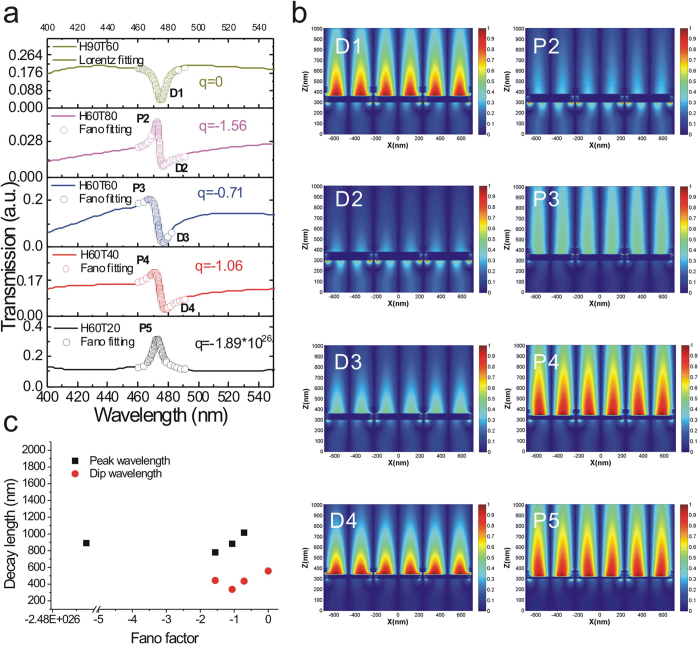
Simulations of transmission spectra and resonance field distributions of the capped nanoslits. (**a**) The calculated transmission spectra of the capped aluminum nanoslits with different structure parameters for normally incident TM-polarized light using the FDTD calculation method. The structure parameters were P = 470 nm, H = 60–90 nm, T = 20–80 nm and W = 60 nm. The open circle lines show Fano and Lorentz fitting curves. The extracted Fano factors were −1.89 × 10^26^, −1.06, −0.71,−1.56 and 0. (**b**) The resonance field (Ez) distributions for the resonance peaks (P2-P5) and dips (D1-D4) with different Fano factors. (**c**) The calculated decay lengths at peak and dip wavelengths for the capped nanoslits with different Fano factors. The average decay lengths were 444 and 892 nm for the resonance dip and peak, respectively. The decay length decreased to 338 nm when the Fano factor was −1.06.

We further utilized finite-difference time-domain (FDTD, FullWAVE 4.0, RSoft) simulations to verify the decreased decay lengths for the Fano resonance at peak and dip wavelengths. Figure 5a shows the calculated transmission spectra of the 470-nm-period capped aluminum nanoslits with different structure parameters for normally incident TM-polarized light. The structure parameters were P = 470 nm, H = 60–90 nm, T = 20–80 nm and W = 60 nm. The Fano resonances with different resonance profiles were observed in the calculated spectra. These resonance profiles match quite well with the measured results as shown in Fig. 4a. We extracted the Fano factors from the Fano and Lorentz fitting curves. They were −1.89 × 10^26^, −1.06, −0.71, −1.56 and 0, respectively. Figures 5b shows the resonance field (Ez) distributions for the resonance peaks (P2-P5) and dips (D1-D4), respectively. Obviously, the Fano resonance dip has a shorter decay length than the resonance peak. The average decay lengths were 444 and 892 nm for the resonance dip and peak, respectively. Besides, the decay length decreased to 338 nm when the Fano factor was −1.06 as shown in Fig. 5c. The calculated results were consistent with the experimental data shown in Fig. 4g. The BW-SPP on the surface has a ~420-nm-long evanescent tail. When the BW-SPP interacts with the cavity mode and a Fano coupling mode occurs. The surface plasmon field is re-distributed. The cavity mode exhibits localized surface plasmon resonance in the nanoslits with a short decay length. It affects the field distribution of the Fano mode and results in a shorter decay length for the coupled resonance. Therefore, we attributed the improved surface sensitivity for the Fano-coupled mode to the decreased decay length.

### Bio-interaction measurements using Fano resonances in capped aluminum nanoslits

**Figure 6:**
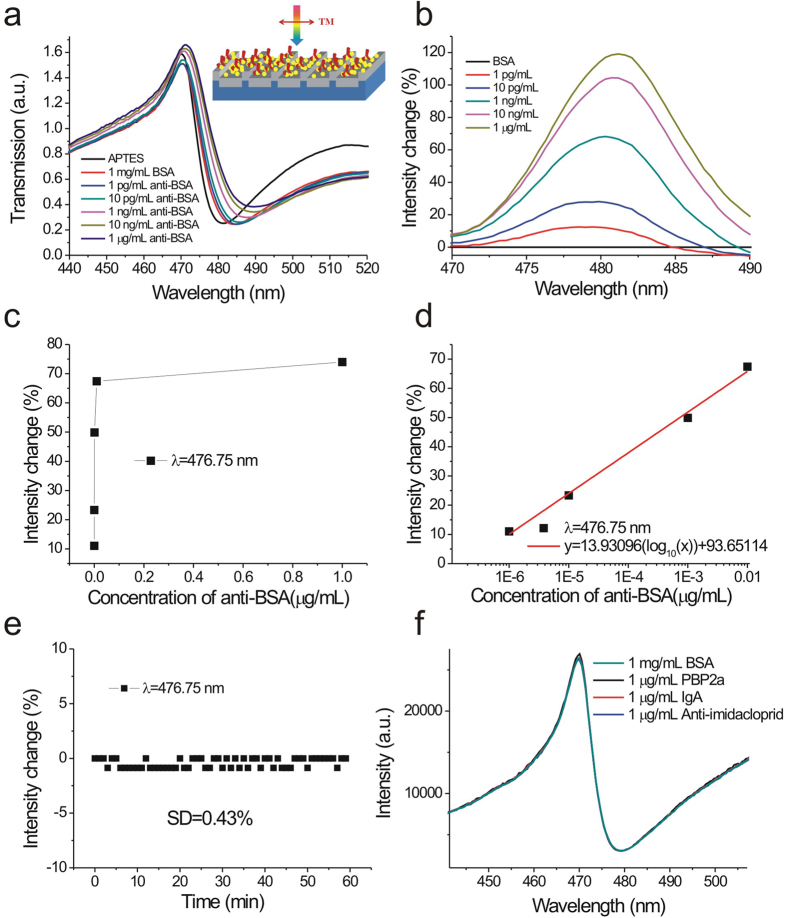
Bio-interaction measurements using Fano resonances in capped aluminum nanoslits. (**a**) The measured transmission spectra in 10% APTES, 1 mg/mL BSA, and different concentrations of anti-BSA solutions from 1 pg/mL to 1 μg/mL for normally incident TM-polarized light. The inset shows a schematic to describe the interactions between anti-BSA and BSA. (**b**) The spectral intensity changes caused by different concentrations of anti-BSA solutions. The transmission spectrum of the BSA solution was set as a reference. (**c**) The intensity change at a wavelength of 476.75 nm as a function of the concentration of anti-BSA. (**d**) The intensity change at a wavelength of 476.75 nm as a function of the logarithm of the concentration of the anti-BSA solution. (**e**) The intensity change at a wavelength of 476.75 nm (*I*_*λ*=*476.75 nm*_) as a function of time. (**f**) The measured transmission spectra in BSA and 3 kinds of antibodies solutions, penicillin binding protein 2α (PBP2α), Immunoglobulin A (IgA), anti-imidacloprid. Compared to the transmission spectrum of the BSA solution, the spectra of PBP2α, IgA, anti-imidacloprid antibodies remain unchanged.

We further studied the limit of detection (LOD) of 470-nm-period capped aluminum nanoslits by detecting different concentrations of anti-BSA solutions with both wavelength and intensity interrogation methods. Figure 6a show the measured transmission spectra in 10% APTES, 1 mg/mL BSA and different concentrations of anti-BSA solutions from 1 pg/mL to 1 μg/mL. The Fano resonances were redshifted as the concentrations increased. We analyzed the spectra and set the transmission spectra of the BSA solutions as references. Obviously, the detectable concentration with wavelength interrogation was below 1 pg/mL. Figure 6b shows the spectral intensity changes caused by different concentrations of anti-BSA solutions. Obviously, even for a concentration of 1 pg/mL, the intensity had a change at a wavelength of 476.75 nm. Figure 6c shows the intensity change at a wavelength of 476.75 nm as a function of the concentrations. The intensity change increased and then gradually saturated as the concentration increased. The responses were 11.0, 23.3, 49.9, 67.4, and 73.9% for 0.000001, 0.00001, 0.001, 0.01, and 1 μg/ml, respectively. There was a linear correlation between the response and the logarithm of the concentration when the concentration was less than 10 ng/mL as shown in Fig. 6d. The calibration curve was described by y = 13.93096(log_10_(x)) + 96.65114, R^2^ = 0.99395. In addition, the measured intensity noise, extracted from the inset in Fig. 6d, was 0.43% (standard deviation of the response). It has been reported that the surface concentration can be determined by the thickness, i.e., the surface concentration (μg/cm^2^) ≈ 0.12 × t (nm)^44^. For 10 μg/mL anti-BSA adsorption, the estimated surface concentration is 0.14 μg/cm^2^ for 1.2 nm thickness^45^. When the measured concentration was 1 pg/mL and the detectable surface concentration was below 0.14 fg/mm^2^. Such a surface concentration was much better than that of the localized plasmon resonance in simple plasmonic nanostructures (1000 pg/mm^2^)^5^, the bulky ATR sensors using an angular detection method (1 pg/mm^2^ with a resolution of 0.1 mdeg)^45^,^46^,^47^,^48^, and the quartz crystal microbalance (QCM) detection technique (20 pg/mm^2^ with a resolution of 0.1 Hz). In addition, based on the calibration curve and current system noise (1.29%, 3 times standard deviations), the LOD of the surface concentration (detectable concentration) of anti-BSA can be estimated by a linear regression equation. This yields a theoretical detection limit of 236 fg/mL (0.03 fg/mm^2^). This LOD was one order of magnitude lower than that of plasmonic metamaterials with topological darkness (0.1 fg/mm^2^ with a phase noise of 5 mdeg)^49^ and graphene-protected copper plasmonics (0.2 fg/mm^2^ with a phase shift of 5 mdeg)^50^. It was noted that though the proposed capped aluminum nanoslit is sensitive. It is based on the immunoassay and relies on the immobilized probes for specific and selective detection of analytes. To verify the specific binding between BSA and anti-BSA and the selectivity of the sensors, we conducted the bioexperiments with three different kinds of antibodies, penicillin binding protein 2α (PBP2α), Immunoglobulin A (IgA), and anti-imidacloprid. Compared to the transmission spectrum of the BSA solution, the spectra of PBP2α, IgA, anti-imidacloprid antibodies remain unchanged (see Fig. 6f). Compared to the widely-used method for immunoassay measurement, enzyme-linked immunosorbent assays (ELISAs), which require the assistance of enzymes and reported antibodies, the SPR sensing provides a simple, real-time and label-free detection technique. In addition to SPR sensing, other label-free sensing techniques without immobilized probes, such as electrochemiluminescent (ECL) sensors, can also provide a sensitive and low-cost way for rapid detections of multiple metal-ions^51^.

## Discussion

We utilized hot embossing nanoimprint lithography and thermal evaporation to fabricate low-cost, large-area, and highly sensitive capped aluminum nanoslits on A4 size plastic films. The intrinsic properties of the aluminum metal, having a large imaginary part of the dielectric function and a longer electromagnetic field decay length, limit the surface sensing capability of nanostructures. It usually has a lower detection sensitivity than gold and silver–based nanostructures. We show that this problem can be overcome by using capped nanoslit structures with optimal structure parameters for the Fano resonance. In our experiments and FDTD simulations, the optimal condition for the Fano factor is ~1.3. It occurs when the thickness of coating aluminum film is similar to the slit height of the imprinted nanostructure. A transverse-magnetic (TM) polarized wave in capped aluminum nanoslits with a period of 470 nm generated extremely sharp and asymmetric Fano resonances in transmission spectra. The bulk (wavelength) sensitivity is independent of Fano factors and mainly determined by the period. Nevertheless, the surface sensitivity is closely related to the Fano factor. The maximum thickness sensitivity is 2.58 nm/nm for wavelength shift and 90%/nm for normalized intensity change. Compared to other Fano factors, the optimal nanostructure increased several times of surface sensitivities. From the FDTD simulations, we attributed the improved surface sensitivity to a reduced decay length. The Fano resonance was formed by the coupling between the slit cavity mode and BW-SPP mode. The cavity mode exhibits localized surface plasmon resonance in the nanoslits with a short decay length. It affects the field distribution of the BW-SPP mode and results in a shorter decay length for the coupled resonance. In our experiments, the BW-SPP mode had a decay length of ~ 420 nm and was reduced to 240 nm when the Fano factor was −1.3. The protein-protein interaction experiments further verified the high sensitivity of the structures and a limit of detection (LOD) of 236 fg/mL. Compared to other SPR sensors utilizing gold films, the proposed aluminum-based nanostructures is cost-effective. On the other hand, to fabricated nanostructure-based SPR sensors, many approaches have been proposed such as interference lithography, nanoimprint lithography, nanosphere lithography, soft lithography techniques, and nanostencil lithography, etc. Among these techniques, nanoimprint lithography is more suitable for fabricating various 2-dimensional or 3-dimensional nanostructures and can solve the problems of mass fabrication, low cost and large area. In addition, as the nanostructures were fabricated on the plastic film using hot embossing nanoimprint lithography, it can be directly integrated to the microfluidic devices made on the plastic film^52^. The sample treatment and plasmonic multiplexed detection can be conducted on a chip, which can contribute to plasmonic biosensors to point-of-care diagnosis^53^. Such low-cost, reproducible and high-throughput fabrication of highly sensitive capped aluminum nanoslits can benefit real sensing applications.

## Methods

### Nanoimprinting process for metallic nanostructures

**Figure 7:**
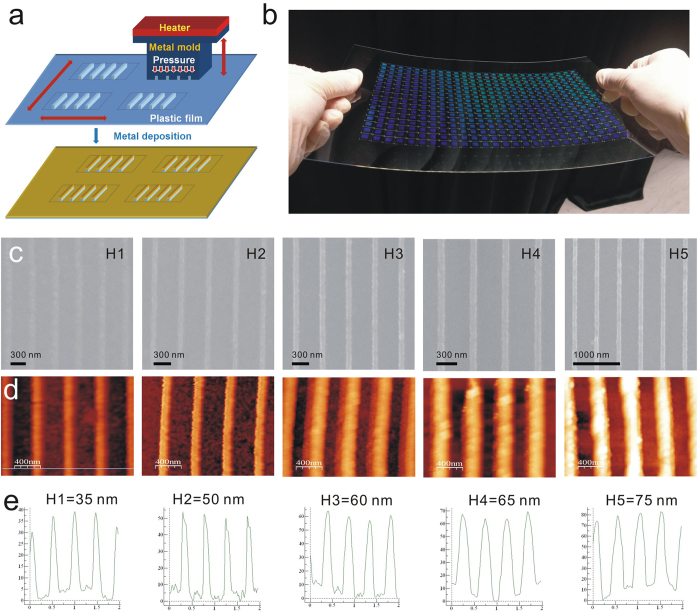
Fabrication of capped aluminum nanoslits using hot embossing nanoimprint lithography and thermal evaporation. (**a**) A process flowchart for the fabrication of capped aluminum nanoslits. (**b**) The optical image of the replicated nanostructure arrays on an A4 size polycarbonate film. There are 416 arrays and the area of each periodic nanostructure is 5 × 5 mm^2^. (**c**) The scanning electron microscope (SEM) images of the capped aluminum nanoslits with different ridge heights. (**d**) The atomic force microscopy images of the capped aluminum nanoslits with different ridge heights. (**e**) The cross-sectional profiles of the capped aluminum nanoslits. The ridge heights range from 35 to 75 nm.

Figure 7a shows a process flowchart for the fabrication of the capped aluminum nanoslits. The nanostructures were produced on a polycarbonate (PC) substrate using hot embossing nanoimprint lithography and thermal evaporation. First, periodic nanogrooves of 60 nm in width, 100 nm in depth and 470 nm in period were fabricated in a resist coated on a silicon wafer using electron beam lithography. A 100-nm-thick diluted ZEP-520 resist (ZEP-520, Zeon Corp, Tokyo, Japan) was spin-coated on a 525-μm-thick silicon substrate. An electron-beam writing system (ELS-F125, Elionix, Japan) was used to draw groove arrays in the resist. The patterns were then sputtered with gold and then electroformed with Ni and Co for producing a 250-μm-thick metal mold. This metal mold was used to replicate the nanostructures on a 178-μm-thick PC film using homemade hot embossing nanoimprint equipment. After imprinted the nanostructures into the plastic film, the mold was peeled off from the replicated plastic film. The nanostructures can be rapidly replicated and the mold can be used repeatedly. With different heating temperatures from 170 to 190 °C, the nanostructures with different ridge heights from 35 to 75 nm were made. After depositing aluminum films with different thicknesses from 42 to 81 nm on the imprinted plastic substrates, the capped aluminum nanoslit arrays were produced. Figure 7b shows the optical image of the replicated nanostructure arrays on an A4 size PC film. There are 416 arrays and the area of each periodic nanostructure is 5 × 5 mm^2^. Figure 1a shows the optical image of the capped aluminum nanoslit arrays. Figure 7c shows the scanning electron microscope (SEM) images of the capped aluminum nanoslits with different ridge heights from 35 to 75 nm.

### Atomic force microscopy (AFM) measurements

All atomic force microscopy images were taken using the Veeco di Innova AFM instrument operating in tapping mode in air. The scan size of the AFM image is typically 5 × 5 μm^2^ at a scan rate of 0.8 Hz. Figure 7d shows the atomic force microscopy images of the capped aluminum nanoslits with different ridge heights. The cross-sectional profiles of the capped aluminum nanoslits are shown in Fig. 7e. The ridge heights are from 35 to 75 nm.

### Optical setup for angular transmission spectrum measurement

A 150 W white light was coupled to a fiber cable and a fiber lens for light collimation. Its incident polarization was controlled by a linear polarizer. The white light was focused on a capped nanoslit array. To control the incident angle, the sample was put on a rotational stage controlled with a stepper motor. The transmission light was collected by another fiber lens and focused on a fiber cable. The angular transmission spectra were taken using a fiber-coupled linear charge-coupled device (CCD) array spectrometer (BWTEK, BTC112E).

### Atomic layer deposition of aluminum oxide

Aluminum oxide films were deposited on the biochips using an atomic layer deposition machine (ALD, Syskey Technology CO., LTD). For deposition of aluminum oxide, the precursors of trimethylaluminum (TMA) and water were used. The chamber working pressure was 1.2 × 10^−1^ torr and the substrate temperature was kept at 120 °C.

### Refractive index sensitivity tests and biosensing experiments

The bulk refractive index sensitivities were measured by pouring purified water mixed with various fractions of glycerin over the sample surface. The refractive indexes of the mixtures (from 0 to 20% glycerin) ranged from 1.3330 to 1.3575. The biosensing experiments were conducted using bovine serum albumin (BSA, Sigma-Aldrich) and anti-BSA (Sigma-Aldrich) assay in a deionized water buffer. First, the nanostructures were exposed to a 10% aminopropyltriethoxysilane (APTES) solution for 30 minutes and then baked at 120 °C for 1 hour to form amino groups on the Al_2_O_3_ film surface. After modification of the amino groups, a 100 μL of 1 mg/mL BSA solution was dropped onto the structure surface for 1 hour. The sample was then washed with ultrapure water to remove the unbound BSA proteins and purged dry by nitrogen gas. Finally, a 100 μL of 1 pg/mL anti-BSA solution was dropped onto the structure surface for 1 hour. The sample was then washed with ultrapure water and purged dry by nitrogen gas. The dropping, washing and nitrogen-purged drying processes were subsequently repeated for different concentrations of anti-BSA solutions from 100 pg/mL to 1 mg/mL. The transmission spectrum measurements were conducted before and after BSA and anti-BSA adsorption.

## Additional Information

**How to cite this article:** Lee, K.-L. *et al*. Highly Sensitive Aluminum-Based Biosensors using Tailorable Fano Resonances in Capped Nanostructures. *Sci. Rep.* **7**, 44104; doi: 10.1038/srep44104 (2017).

**Publisher's note:** Springer Nature remains neutral with regard to jurisdictional claims in published maps and institutional affiliations.
